# Postpartum hemoperitoneum due to rupture of a blood vessel on a uterine pseudo tumor: a case report

**DOI:** 10.11604/pamj.2013.16.57.3363

**Published:** 2013-10-17

**Authors:** Gregory Halle Ekane, Pierre Marie Tebeu, Thomas Egbe Obinchemti, Theophile Nana Njamen, Charlotte Tchente Nguefack, Jacques Tsingaing Kamgaing, Eugene Belley Priso

**Affiliations:** 1 Department of Gynaecology & Obstetrics, University of Buea, Cameroon; 2 Department of Gynaecology & Obstetrics, Douala General Hospital, Douala, Cameroon; 3 Department of Gynaecology & Obstetrics, University Teaching Hospital, Yaounde, Cameroon; 4 Ligue d'Initiative et de Recherche Active pour la Santé et l'Education de la Femme, Cameroon

**Keywords:** Postpartum, hemoperitoneum, pseudotumor, pregnancy, myomas

## Abstract

We are reporting a case of hemoperitoneum followed by early post partum collapse due to bleeding from a ruptured vessel on the surface of an undiagnosed uterine pseudo tumor. There are literature reports of spontaneous hemoperitoneum from bleeding of superficial vessels over lying myomas during pregnancy but a case of rupture of a blood vessel on a uterine pseudo tumor leading to isolated hemoperitoneum in the immediate postpartum period is a rare event. We are presenting the literature review and some aspects of the management of this case. The importance of having a high index of suspicion in cases of hemoperitoeum occurring immediately after delivery especially in a low income setting where radiologic imaging techniques like magnetic resonance imaging (MRI); which is the most sensitive diagnostic tool in cases of ruptured vessels are rare is highlighted.

## Introduction

Postpartum hemorrhage (PPH) remains the most important cause of maternal death in both the developed and the low-income countries [1]. Severe PPH is characterized by vaginal bleeding and alterations of the vital signs. However, bleeding might be concealed in some forms of PPH, therefore little bleeding is observed vaginally. In such cases, the major clinical findings are alteration of the vital signs and sudden increase of the abdominal circumference with shifting dullness and a fluid thrill that characterizes the hemoperitoneum. Hemoperitoneum is a potential life-threatening condition. It can also occur during pregnancy [2–4] or after a normal vaginal delivery [5]. It requires urgent management either by laparotomy or laparoscopy depending on the hemodynamic condition of the patient [6]. The diagnosis of postpartum hemoperitoneum after a vaginal delivery with overt vaginal blood loss of less than 500 ml requires a high index of suspicion to reduce the morbidity and/ or mortality associated with this clinical entity. We are presenting a case of severe PPH with hemoperitoneum in the early postpartum period diagnosed clinically and confirmed by ultrasound that was managed by laparotomy.

## Patient and observation

A 34 year-old Gravida 5, Para 1 was received at the Douala General Hospital with a 39 weeks gestation calculated from the last menstrual period for antenatal evaluation. Her medical records showed two induced abortions by dilatation and curettage. Her antenatal visits had been uneventful. After this last visit the patient was sent home but returned five days later to our maternity where she was diagnosed to be in labour and admitted into the labour room. She later on had a normal vaginal delivery of a life born female baby weighing 3250gm with APGAR score of 9 and 10 at the first and fifth minute respectively. Delivery of the placenta by controlled cord traction was initially unsuccessful so the Crede Maneuver was employed. The membranes and the placenta were complete and the uterus was well contracted. The estimated blood loss was about 250ml. She was hemo-dynamically stable immediately after delivery.

However, an hour after delivery the patient started complaining of weakness, dizziness and shortness of breath. She became agitated with signs of hypovolemic shock. Immediate resuscitative measures were started. Her lung fields were clear. The abdomen was full and a slightly tender at the hypogastric region. The uterus was about 20 week's size (level of the umbilicus) and well contracted. On vaginal examination, there was slight vaginal bleeding but no lacerations or tears. A digital exploration of the uterine cavity did not reveal clots, placenta debris or a uterine defect suggestive of uterine rupture. The clinical diagnosis of a hemoperitoneum was made.

Her hemoglobin level dropped to 7g/dl as compared to 11.2g/dl on admission 75 minutes after delivery so she was resuscitated with intravenous macromolecules and transfused 1000ml of whole blood as initial emergency measures. Furthermore, a two way Foley's catheter was also inserted in the urinary bladder. Her coagulation profile and platelet count were normal. Ultrasonography revealed an intra abdominal fluid collection. The uterus was enlarged but there was no evidence of retained products of conception. There were no adnexal masses. An ultrasonograhic guided abdominal paracentesis, confirmed the presence of a hemoperitoneum.

**Figure 1:**
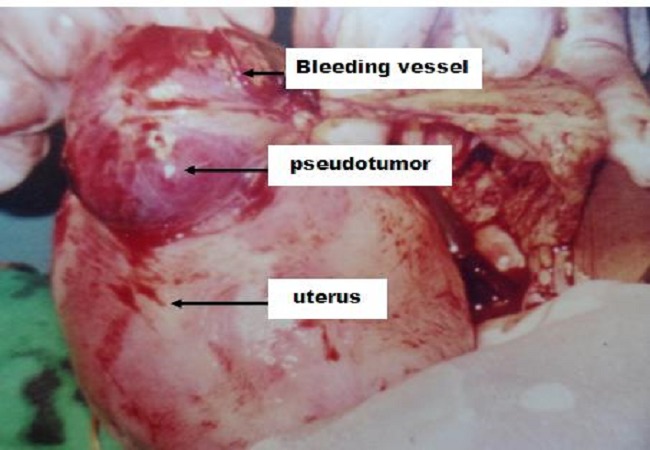
Image showing the ruptured vessel overlying the pseudo tumor with omentum adherent to it

A consent form was duely signed by the patient's relatives and 45 minutes later she underwent an exploratory laparotomy after she had been stabilized. Per-operatively, two liters of blood was suctioned from the peritoneal cavity. The uterus was well contracted with no evidence rupture. However, there was a firm, well vascularized mass at the uterine fundus measuring 6,0 cm x 5,0 cm with two large bleeding vessels on its surface. A portion of omentum was adherent to the mass (Figure 1). The other abdominal organs were normal.

**Figure 2:**
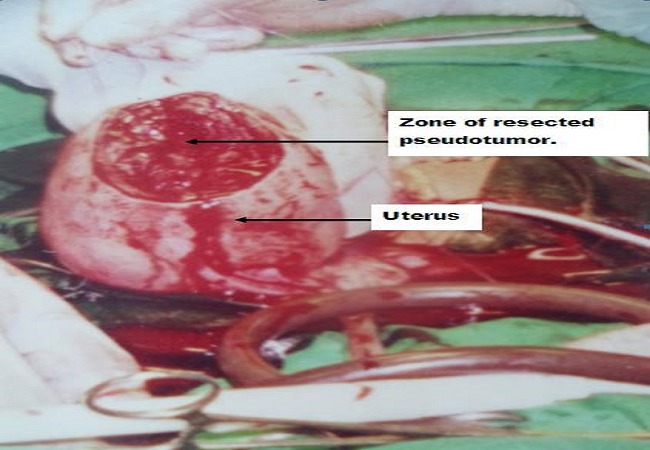
Zone of resected pseudo tumor at the uterine fundus

The mass was resected with a small infraction of the uterine cavity since the mass was mainly subserous and a small portion intra-mural (Figure 2). The site resection of the uterus was repaired in two layers with absorbable Polyglycan sutures (vicryl 1^®^ Ethicon France). She remained stable after transfusion of 1000ml of blood per-operatively. The post operative period was uneventful and she was discharged from hospital on the fifth postoperative day with her newborn. The histologic analysis of the resected mass revealed a few granulomatous, collagenous and fibrous tissues with areas of calcification. The patient was reviewed two weeks after discharge from the hospital. Her general condition was satisfactory and the serum beta hCG assay was 15 IU/L.

## Discussion

Postpartum collapse due to severe PPH is a life threatening condition if resuscitative and therapeutic measures are not promptly instituted. Often when the patient is well evaluated the cause of PPH can quickly be elucidated as this is usually due to uterine atony, genital lacerations, or consumptive coagulopathy [7]. However, intra-peritoneal bleeding after normal vaginal delivery though very rare with an undefined etiology can also cause postpartum collapse. Its occurrence poses both diagnostic and therapeutic difficulties. In early pregnancy the commonest cause of hemoperitoneum is a ruptured ectopic pregnancy. Immediately after delivery with the existence of after labor pains, abdominal pain which might be suggestive of an intra-abdominal pathology can be masked. Our patient only complained when there was a rapid change in her hemodynamic status. On evaluation, there were no signs suggestive of pulmonary embolism. Abdominal tenderness which was not associated with active profuse vaginal bleeding but with signs of hypovolemic shock was suggestive of a hemoperitoneum secondary to the rupture of a vessel or an abdominal organ. Pre-operative assessment did not permit the exact localization of the lesion. A similar case was reported by Hung-Chung et al where multi row computed tomographic angiography was used to identify the cause of hemoperitoneum [8]. This diagnostic technique is not available in our setting like in many hospitals in low income countries. Secondly, even with the initial resuscitative measures, the patient remained unstable so this would not have been appropriate. An ultrasonographic guided abdominal paracentesis confirmed the hemoperitoneum which necessitated an exploratory laparotomy. Some authors used transabdominal and transvaginal Doppler sonography as a diagnostic tool in cases of pseudo aneursyms causing PPH [9, 10]. With regards to our patient, in the presence of hemoperitoneum and considering the size of the bleeding vessel, it would have been difficult to locate the source of bleeding using a transabdominal Doppler ultrasonography.

At laparotomy, a highly vascularized uterine pseudo tumor was diagnosed with ruptured vessels on its surface. One is tempted to attribute this rupture to the Crede Maneuver and uterine message which were used during the management of the third stage of labor. The occurrence of hemoperitoneum which is probably linked to carrying out of the above procedures in the presence of an undiagnosed highly vascularized serosal uterine mass emphasizes the fact that pressure on the uterus should be exerted with caution during the management of third stage of labor.

Choriocarcinoma presenting with metastasis can be associated with vascular masses protruding on the surface of the uterus or other organs can be a rare cause of hemoperitoneum in the postpartum period [11]. Histological examination revealed a mass made up of fibrous and granulomatous tissue at the site that looked like a zone of previous uterine perforation after repeated dilatation and curettage. Chorionic villi were not documented in the histological specimen and a serum Beta hCG assay was very low, this excluded the possibility of the pseudo tumor being a form of gestational trophoblastic disease.

## Conclusion

In cases of postpartum collapse the existence of life threatening hemoperitoneum with minimal vaginal bleeding should be excluded. A high index of suspicion of this clinical entity is imperative so as not to delay diagnosis and management. Bleeding can be stopped by carrying out an exploratory laparotomy even in a low income setting.

## References

[1] Tebeu PM, Fezeu LY, Ekono MR, Kegne FG, Folifack YF, Fomulu JN (2013). Post partum haemorrhage at the Yaounde University Hospital, Cameroon. Int J Gynaecol Obstet..

[2] Miro Kasum (2010). Hemoperitoneum caused by a bleeding myoma in pregnancy. Acta Clin Croat.

[3] Wong L, Ching TW, Kok TL (2005). Spontaneous hemoperitoneum from a uterine leiomyoma in pregnancy. Acta Obstet Gynecol scand..

[4] Aziz U, Kulkarni A, Lazic D, Cullimore E (2007). Spontaneous rupture of the uterine vessels in pregnancy. Acta Obstet Gynecol Scand..

[5] Dalal S, Garg P, Jain A, Nityasha (2007). Spontaneous hemoperitoneum After Normal Vaginal Delivery: A Case Report & Brief Review of Literature. The Internet Journal of Gynecology and Obstetrics.

[6] Galeo JL, Lortie K, Singh SS (2010). Laparoscopic internal iliac artery ligation for post partum spontaneous hemoperitoneum. J Obstet Gynaecol Can..

[7] Society of Obstetrics and Gynecology of Canada (2008). Postpartum hemorrhage. ALARM Manual.

[8] Hung-Chung Fu, Sheung-Fat Ko, Chia-Yu Ou, Te-Yao Hsu (2006). Postpartum haemoperitoneum due to alvusion of pelvic uterine adhesion band. Sounthern Medical Journal.

[9] Cooper BC, Hocking-Brown M, Sorosky JI, Hansen WF (2004). Pseudo aneurysm of the uterine artery requiring bilateral uterine artery embolization. J Perinatol..

[10] Langer JE, Cope C (1999). Ultrasonographic diagnosis of uterine pseudoaneurysm affer hysterectomy. J Ultrasound Med..

[11] Erb RE, Gibler WB (1989). Massive hemoperitoneum following rupture of hepatic metastases from unsuspected choriocarcinoma. Am J Emerg Med..

